# Selection on the mitochondrial *ATP synthase 6* and the *NADH dehydrogenase 2* genes in hares (*Lepus capensis* L., 1758) from a steep ecological gradient in North Africa

**DOI:** 10.1186/s12862-017-0896-0

**Published:** 2017-02-07

**Authors:** Hichem Ben Slimen, Helmut Schaschl, Felix Knauer, Franz Suchentrunk

**Affiliations:** 1 UR Génomique des Insectes Ravageurs des Cultures d’Intérêt Agronomique (GIRC), Université de Tunis El-Manar, 2092 El Manar, Tunisia; 2 Institut Supérieur de Biotechnologie de Béja, Beja, 9000 Tunisia; 3 Department of Anthropology, University of Vienna, Althanstrasse 14, 1090 Vienna, Austria; 4 Research Institute of Wildlife Ecology, University of Veterinary Medicine Vienna, Savoyenstrasse 1, 1160 Vienna, Austria

**Keywords:** Mitochondrial DNA, Positive selection, Environmental variation, Proteins, Hares, Tunisia

## Abstract

**Background:**

Recent studies of selection on mitochondrial (mt) OXPHOS genes suggest adaptation due mainly to environmental variation. In this context, Tunisian hares that display several external phenotypes with phylogenetically rather homogenous gene pool and shallow population structure provide a good precondition to detect positive selection on mt genes related to environmental/climatic variation, specifically ambient temperature and precipitation.

**Results:**

We used codon-based methods along with population genetic data to test for positive selection on ATPase synthase 6 (ATP6) and NADH dehydrogenase 2 (ND2) of cape hares (*Lepus capensis*) collected along a steep ecological gradient in Tunisia. We found significantly higher differentiation at the ATP6 locus across Tunisia, with sub-humid Mediterranean, semi-arid, and arid Sahara climate than for fourteen unlinked supposedly neutrally evolving nuclear microsatellites and mt control region sequences. This suggested positive selection on ATP6 sequences, which was confirmed by several codon-based tests for one sequence site that together with a second site translated into four different amino acids. Positive selection on ND2 sequences was also confirmed by several codon-based tests. The corresponding frequencies of the two most prevalent variants at each locus varied significantly across climate regions, and our logistic general linear models of occurrence of those proteins indicated significant effects of mean annual temperature for ATP6 and mean minimum temperature of the coldest month of the year for ND2, independent of geographical location, annual precipitation, and the respective co-occurring protein at the second locus. Moreover, presence of the ancestral ATP6 protein, as inferred from phylogenetic networks, was positively affected by the simultaneous presence of the derived ND2 protein and *vice versa*, independent of temperature, precipitation, or geographic location. Finally, we obtained a significant coevolution signal for the ancestral ATP6 and derived ND2 sequences and *vice versa.*

**Conclusions:**

positive selection was strongly suggested by the population genetic approach and the codon-based tests in both mtDNA genes. Moreover, the two most prevalent proteins at the ATP6 locus were distributed at significantly varying frequencies across the study area with a significant effect of mean annual temperature on the occurrence of the ATP6 proteins independent of geographical coordinates and the co-occuring ND2 protein variant. For ND2, occurrence of the two most frequent protein variants was significantly influenced by the mean minimum temperature of the coldest month, independent of the co-occurring ATP6 protein variant and geographical coordinates. This strongly suggests direct involvement of ambient temperature in the adaptation of the studied mtOXPHOS genes.

**Electronic supplementary material:**

The online version of this article (doi:10.1186/s12862-017-0896-0) contains supplementary material, which is available to authorized users.

## Background

The mammalian mitochondrial (mt) DNA, a circular double-stranded molecule, contains 37 genes, including thirteen protein coding genes, 22 transfer (t)RNA genes and two ribosomal (r)RNA genes. All protein coding genes are components of the mt respiratory chain (OXPHOS—oxidative phosphorylation): these are seven subunits of the NADH dehydrogenase complex I (ND1, ND2, ND3, ND4, ND4L, ND5, and ND6), one subunit (cytochrome *b*) of the cytochrome c*b*1 complex III, three subunits of cytochrome *c* oxidase complex (CO1, CO2, and CO3), and two subunits of ATPase complex V (ATP6 and ATP8) [1].

Particularly the high copy number in cells, its maternal inheritance, its high point mutation rate, the fact that recombinations are evidently occurring very rarely in mammals and other animals, as well as its compact architecture and small total size have made mtDNA very popular for phylogenetic, phylogeographic, and population genetic studies for a wide range of taxa. The majority of these studies, however, implicitly considers mtDNA as neutrally evolving and mtDNA variability is usually interpreted to reflect the effects of gene flow and random drift, whereas molecular changes are supposed not to influence the fitness of the individuals.

Whether mainly neutral evolutionary forces or adaptive evolution are shaping mtDNA variation is contentiously debated (e.g., [2–5]). Whereas there seems to be no major discussion on the fact that strong purifying selection is considerably affecting mtDNA variability of mtOXPHOS genes in animals (e.g., [2, 3, 6, 7]), mechanisms of how positive selection may shape spatio-temporal patterns of such genes are hardly explored. Evidence of positive selection on segments of mitogenomes of various animal species has only relatively recently started to accumulate in the literature [8–16]. Moreover, as to our knowledge, even no preliminary view is at hand of how positive selection of coevolving mtOXPHOS genes might influence intraspecific mitogenome variability under different environmental contexts.

Despite strong purifying selection already during the transmission of mtDNA down the maternal lineages (e.g., [3]), less pathogenic (deleterious) mutants harboured by heteroplasmic mothers and inherited to the next generation may result in retarded growth, disorders, or serious health problems in the offspring (e.g., [17, 18]). Over 100 point mutations in protein coding genes, tRNAs, and ribosomal rRNAs of the human mitogenome have been reported to be associated with a wide range of clinical symtoms or diseases [19]. The majority of mtDNA mutations that cause disease in humans are, however, considered mild to moderately deleterious and serious mutations are apparently effectively selected against along the maternal germ line, as suggested by the mouse model [20].

Although some [15] suggested that a molecular signature of positive selection does not necessarily imply adaptation several hypotheses have been proposed to explain selection on mtDNA. They all imply adaptation to different environmental conditions such us high altitude, climate change, temperature, colder climate, food availability, and habitat change (see [21] for overview). Hares of the genus *Lepus* were suggested to be a suitable group to study natural selection in mitogenomes given their recent evolutionary history and their wide distribution in contrasting environments [14]. Analyses of eleven mitogenomes of different hare species of temperate and arctic origins by the latter authors have suggested positive selection on several codons of genes of the mtOXPHOS complexes, most notably affecting the arctic hare lineage. However, the structure and the physicochemical properties of the encoded proteins seemed to be not affected by these amino-acids substitutions. The authors [14] concluded that the functional impact of the amino acid changes may have been underestimated by their analysis. Moreover, [22] have suggested that codon sites showing functional changes might not show a high ω value. By contrast, the codon sites where many amino acid substitutions occurr may be falsely predicted as positively selected sites because of a high ω value that is obtained by chance even if the substitutions were essentially neutral. Therefore, in the absence of an appropriate statistical tool or experimental analyses, an adaptive or non-adaptive value of the positively inferred selected sites remains elusive.

In this study we test for selection on two mtOXPHOS genes, *ATP synthase 6* and *NADH dehydrogenase 2*, in hares from a small region in North Africa (Tunisia) where climatic and habitat conditions exhibit a dramatic clinal change from the Mediterranean seaboard in the north to the arid Sahara in the south along a short geographic distance (less than 500 km), but without obvious physiographic barriers to gene flow. In fact, earlier population genetic and phylogenetic investigations [23] revealed shallow differentiation in hares across this region on the one hand and the presence of only one hare species (i.e., cape hare, *Lepus capensis* L, 1758; see also [23–25]) on the other. For our current analyses we use the same individuals that have already been analysed in population genetic and phylogenetic terms; hence, the currently observed mtOXPHOS gene variation has been obtained from the same populations of mitochondria that have already revealed very shallow genetic divergence across the different climate zones in Tunisia. Under positive selection on those two loci and adaptation to different environments we expected significant changes of protein frequencies across the study region in addition to site-specific selection signals for the corresponding sequences. Furthermore, we expected significant effects of ambient temperature or precipitation, as important climatic characteristics, on the occurrence of the most prevalent proteins encoded by the two loci. Finally, we expected a significant signal of co-evolution of the two loci under study, particularly because the non-recombining mtDNA can be considered as one linkage system. We observed co-evolution of the two loci, but positive selection on combinations of ancestral and derived proteins for ATP6 and ND2, and *vice versa*, in addition to a signal of positive selection on single positions of ATP6 and ND2, as well as ambient temperature effects on the occurrence of the most prevalent proteins.

## Methods

### Specimen samples and ecological characteristics of the study area

**Fig. 1:**
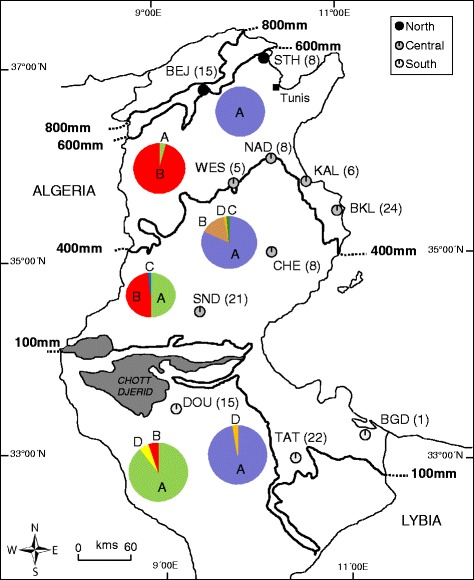
Sampling regions of hares from North, Central and South Tunisia. Sample sizes appear in parentheses. Hares were grouped into three populations according to climatic, geographic and phenotypic data. The north population with samples from two regions (BEJ: Béja and STH: SidiThabet); the central population with samples from six regions (NAD: Nadhour; WES: Weslatia; KAL: Kalâa; BKL: Bekalta; CHE: Cherarda; SND: Sned); and the south population with samples from three regions (DOU: Douz; TAT: Tataouine, and BGD: Ben Guerdène). Pie charts indicates protein frequencies (see Fig. 2 for protein names) for ATP6 (left) and ND2 (right) in the three populations

A total of 133 hares conventionally considered cape hares (*Lepus capensis*, e.g., [24, 26–28]) were collected at eleven sampling sites in Tunisia between 2003 and 2006. The sampling localities ranged between close to the northern Mediterranean seaboard at N 36° 55′ 17″ and the Sahara at N 32° 52′ 34′. Acronyms of sampling localities, geographic coordinates, positions within northern (NT), central (CT), and southern (ST) Tunisia, as well as sample sizes are given in Fig. 1. The whole sampling area expanded from the Mediterranean humid and sub-humid bioclimatic zones in the north (NT), characterized by Mediterranean forest and scrubland (maquis), across the semi-arid central part of Tunisia (CT), with steppe vegetation including chenopods (*Arthemisia*, *Ephedra*), to the arid and hyper-arid Sahara in the south (ST), with typical desert vegetation (e.g., [29]). It represents a remarkably steep climatic and ecological gradient across a straight distance of less than 450 km, with means of annual precipitation and temperature ranging between 477 mm and 17.1 °C in the north and 52 mm and 21.3 °C in the south, and aridity indices between 37.8 in the north and 2.9 in the south. Whereas the coefficient of variability of the aridity indices for Tunisia is amongst the highest of all African countries, mean annual temperatures do not vary to such an extent across whole Tunisia [30] and even less across our study area.

In addition to the Tunisian samples, single specimens of *Lepus capensis* from South Africa (Overberg Region, Western Cape), Egypt, west of the Nile (Matruh), Egypt, east of the Nile (Sinai), as well as single specimens of scrub hare, *L. saxatilis*, (Western Cape, R.S.A.), and one brown hare, *L. europaeus*, from eastern Austria (Zwerndorf) were included for outgroup comparison in the phylogenetic analyses of the Tunisian ATP6 and ND2 sequences/proteins (see below).

### DNA extraction and PCR amplification

Total genomic DNA was extracted using the GenElute Mammalian Genomic DNA Miniprep kit (Sigma-Aldrich) from diverse tissues (liver, skelettal muscles, ear cartilage, tongue, see [26, 31] of each specimen. PCR amplification of two mitochondrial (mt) protein coding sequences were performed according to [32]: 1) a 453 bp segment of the mt ATP synthase 6 (ATP6) gene from site 8142 to 8594 of the complete mt genome of the brown hare, *Lepus europaeus* [33]; and 2) a 409 bp segment of the mt NADH dehydrogenase 2 (ND2) gene from site 4066 to 4474 [33]. Purification of amplicons was carried out using the Wizard SV gel and PCR clean-up system (Promega) DNA purification Kit. Forward and reverse strand sequencing was performed by Macrogen Inc., South Korea. In addition, sequences of the hypervariable domain 1 of the mt control region (HV1) and genotype data of fourteen microsatellite loci of the same 133 specimens [23] were included as non-coding markers to estimate levels of most likely neutral mitochondrial and nuclear genetic differentiation across the study region.

### Sequence analysis and diversity

Sequences were edited and aligned by eye using the BioEdit v.7.2.5 program©, 1997-2013 [34]. The following indices of DNA polymorphism were estimated by using DnaSP v.5 [35]: haplotype diversity (h), nucleotide diversity (π), the mean number of pairwise differences (k), and the number of segregating (polymorphic) sites (S). All indices were calculated separately for the combined populations of north (NT), central (CT), and south Tunisia (ST), respectively.

### Phylogenetic relationships of haplotypes

To reveal phylogenetic relationships of ATP6 and ND2 sequences and the translated proteins, median-joining (MJ) networks [36] were constructed, including all currently produced haplotypes based on equally weighted variable positions, by using Network 4.2.0.1 (available at http://www.fluxus-technology.com/sharenet.htm). That approach allowed for the detection of possible alternative intraspecific pathways of protein evolution with the chance of identifying possible parallel protein evolution. Our networks were phylogenetically rooted by the above mentioned outgroup sequences of *Lepus capensis*, *L. europaeus*, and *L. saxatilis*.

### Detecting positive selection

To detect positive selection at single amino acid sites, the maximum likelihood (ML) method [37] implemented in CODEML of the PAML 3.14 package [38] was used. Depending on ω values that represent the ratio of the rates of non-synonymous to synonymous nucleotide substitution, sites are expected to evolve neutrally (ω = 1) or under positive (ω > 1) or purifying (ω < 1) selection. Different codon-based models that allowed for variable selection among sites as recommended by [39] (see also e.g., [14, 15]) were tested: scenarios where non-synonymous mutations were neutral or deleterious (models M1a and M7, respectively) were compared with models that allowed for positive selection including an additional category for advantageous substitutions (M2a, M3, M8). Pairwise comparisons of nested models were performed using the likelihood ratio test (LRT): twice the log-likelihood difference was compared with a *χ* ^2^ distribution with degrees of freedom equal to the difference in the number of parameters between both models. In this way, the more general models M2 and M3 could be tested against M1 and M8 against M7. When the LRT was significant, a Bayes Empirical Bayes (BEB) method was used to calculate the posterior probabilities of codon classes in models M2a and M8. Posterior probabilities of > 0.95 were considered as supported under the BEB method [40].

As alternative methods and in order to identify codons affected by positive and negative selection, fast single-likelihood ancestor counting (SLAC), fixed effects likelihood (FEL), random effects likelihood (REL) and mixed effects model of evolution (MEME) methods available in the HyPhy package [41] and in a free public web implementationon http://www.datamonkey.org (last accessed 15 March 2016; [42]) were applied to our ATP6 and ND2 sequences using the best fit nucleotide model for each data set. The best fitting model was HKY85 for both mtDNA coding regions, as suggested by the program. After reconstructing ancestral sequences, SLAC compares normalized expected and observed numbers of synonymous and nonsynonymous substitutions per variable site. FEL compares the instantaneous synonymous site rate (α) and the instantaneous nonsynonymous site rate (β) on a per site basis, without assuming a prior dN/dS distribution. REL allows for heterogeneity both in synonymous and nonsynonymous rates, by fitting discrete distributions of rates across sites and then inferring the rate per site. MEME estimates the probability for a codon to have undergone episodes of positive evolution, allowing the ω ratio distribution to vary across codons and branches in the phylogeny. A detailed account of the SLAC, FEL, and REL methods is given by [43]. Details of the MEME method are provided by [44], who recommended this method for identifying sites under selection. Significance levels of *P <* 0.05 in SLAC, FEL, MEME and Bayes factors > 50 in REL were considered as indication of positive selection. All tests described above were applied to single genes and to the concatenated sequences.

### Genetic differentiation

In addition to the ATP6 and ND2 data, 461 bp long sequences of the hyper-variable part 1 of the mt control region (mtHV-1) and genotypes of 14 microsatellites obtained earlier for the same individuals [23] were used to estimate genetic differentiation between the three study regions (NT, CT, ST). Generally, under positive selection, one would expect higher differentiation in more conservative markers (i.e. ATP6 and ND2) compared to rapidly evolving ones (i.e. microsatellites and mtHV-1) even in the same linkage system. Therefore, a higher level of gene flow in the fast evolving microsatellites and mtHV-1 sequences than in the ATP6 and ND2 sequences would corroborate positive selection in the latter two genes. In such case, selection would counteract gene flow by shaping mtDNA sequence polymorphisms of ATP6 and ND2 in the different populations.

Therefore, the amount of variation due to partitioning into the presently considered three regions (NT, CT, ST) was calculated by AMOVA models using ARLEQUIN 3.1 [45], separately for ATP6, ND2, mtHV1sequences, and the microsatellite loci. The same program was used to quantify the degree of differentiation between and across regions by calculating pairwise F_ST_. Calculations were carried out separately for each mtDNA domain based on haplotype sequences, and also based on the translated amino acid sequence frequencies for the ATP6 and ND2 genes. Pairwise F_ST_ values for microsatellite loci were calculated using the program Genetix 4.05 [46], and significance levels were adjusted by strict Bonferroni corrections for multiple comparisons after 1000 permutations.

### Tests of protein frequencies across the three regions

Frequencies of the two most prevalent proteins at either locus (that comprised most of all detected haplotypes per locus, respectively) were tested for significant differences between regions by three pairwise Fisher’s exact tests using SPSS 6. Thereby, significance values (α < 0.05) were adjusted by strict Bonferroni correction to account for three tests per locus.

### Test of protein association and coevolution

To test for a significant association of proteins encoded by the ATP6 and ND2 genes within individual mitochondria, Pearson’s phi coefficient of association (φ) was calculated for the two most prevalent proteins in hares from the CT region only, as protein variation was very low in the other two regions (NT, ST) (see Results). This coefficient represents a measure of co-occurrence of two binary variables and is similar in its interpretation to Pearson’s correlation coefficient. It could be applied to the current data set, as only two proteins were revealed at high frequencies at each locus.

To specifically test for coevolution of ATP6 and ND2 sites we used the codon-based Bayesian SPIDERMONKEY approach implemented in DATAMONKEY for our concatenated ATP6 and ND2 sequences from CT. This approach reconstructs the substitution history of the amino acid alignment by maximum likelihood-based methods and analyzes the joint distribution of substitution events using Bayesian graphical models to identify significant associations among sites [47].

### Testing for effects of climate variables on protein occurrence and for co-occurrence of protein variants

Given the reported effects of ambient temperature on mitochondrial protein-coding sequences in humans and fish [8, 13, 48], a significant effect of ambient temperature on the presence of the presently detected protein variants would corroborate selection signals at the ATP6 and ND2 loci as obtained from our molecular test statistics. Thus, we used the statistical software package R 2.15.0 (R Development Core Team, 2011) to run logistic generalized linear models (glm) and model averaging statistics separately for the ATP6 and ND2 proteins, to test for effects of climate indices on the occurrence of the two most prevalent protein variants at each locus. Due to existing uncertainty in model selection, we used techniques of model-averaging and multimodel inference ([49]; R package MuMIn [50]). Thereby, model evaluation is based on the Akaike Information Criterion corrected for small sample sizes (AICc) and all models (estimates and standard errors) are averaged based on the probability (so-called Akaike weight) of this particular model being the best of all models explaining the data. This gives unconditional estimates (variable or factor coefficients and *P-*values). Based on the Akaike weights, we calculated the relative variable importance (RVI) for each variable, i.e., the probability of a particular variable to be present in the best model. This statistical approach is increasingly being applied to circumvent ambiguities in model selection (e.g., [51, 52]). For our models we used climate statistics from the nearest weather stations to our sampling sites [30]. Specifically, we used “mean annual temperature“ (temp), “mean minimum temperature of the coldest month of the year“ (mintemp), and “mean annual precipitation“ (prec) as predictive variables in addition to geographical position (“geographical latitude“– lat; “longitude“- long), as well as the protein variant (A = “ancestral” or B = “derived”) encoded by the respective alternate locus in the same individual. To facilitate the interpretation of the resultant factor coefficients, codes of the two respective protein variants for each locus were concordant (protein variant A = “ancestral”, encoded as 1; protein variant B = “derived”, encoded as 2).

First we run two logistic glms for presence/absence of the two most prevalent proteins (A – phylogenetically ancesral protein, B – phylogenetically derived protein) separately for ATP6 and ND2, to prove whether “mean annual temperature“ (temp) or “mean minimum temperature of the coldest month of the year“ (mintemp) had the highest explanatory value, given all other factors and variables in the models were identical. As “temp” resulted in a significantly more likely model than “mintemp“for the ATP6 protein model (AICc for the “temp“model = 118.75 vs. AICc for the “mintemp“- model = 122.62), the variable “temp“ was used in all further ATP6 protein models. For the ND2 protein models, however,”mintemp“was used for further modelling, as it resulted in a significantly more likely initial model than when using “temp“ (AICc for the “mintemp“ model =62.63 vs. AICc for the “temp“model = 74.03).

Consequently, the syntaxes of our statistical protein models were:

$$ \mathrm{m}=\mathrm{g}\mathrm{l}\mathrm{m}\left(\mathrm{atpab}\hbox{-} 1\sim \mathrm{nd}2\mathrm{ab}+\mathrm{lat}+\mathrm{long}+\mathrm{prec}+\mathrm{temp}\right) $$

and

$$ \mathrm{m}=\mathrm{g}\mathrm{l}\mathrm{m}\left(\mathrm{nd}2\mathrm{ab}\hbox{-} 1\sim \mathrm{atpab}+\mathrm{lat}+\mathrm{long}+\mathrm{prec}+\mathrm{mintemp}\right) $$

## Results

### Genetic diversity of ATP6 and ND2 mtDNA genes

**Fig. 2:**
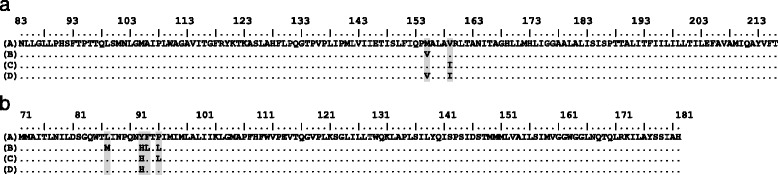
Amino acid sequence alignement of ATP6 (**a**) and ND2 (**b**) haplotypes. A to D are the names of the different proteins detected in each gene. Numbering of amino acid positions is based on the full gene sequence in *L. europaeus* (AJ421471 [33]). Shaded columns represent positions **a** 72 and 76 for ATP6; and **b** 16, 22, 23 and 25 for ND2

**Fig. 3:**
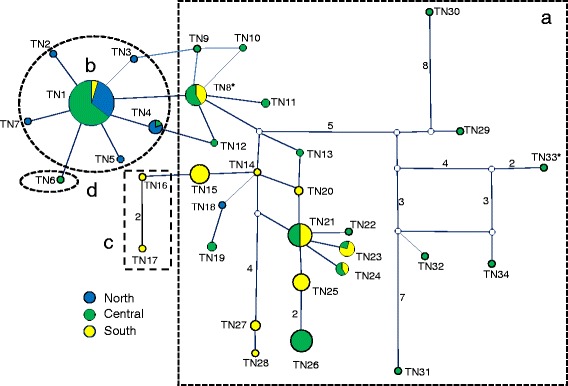
Median-joining network showing the relationships among ATP6 haplotypes. Relative haplotype frequencies correspond to haplotype circle size (see Table 1). Numbers on lines connecting haplotypes indicate numbers of mutation changes. Small white circle indicates inferred haplotype. Blue circle: North population, green circle: central population, and yellow circle: south population. **a**, **b**, **c** and **d** are the different amino acids obtained for ATP6 (see Fig. 2). *: haplotype linked to outgroup sequences

**Fig. 4:**
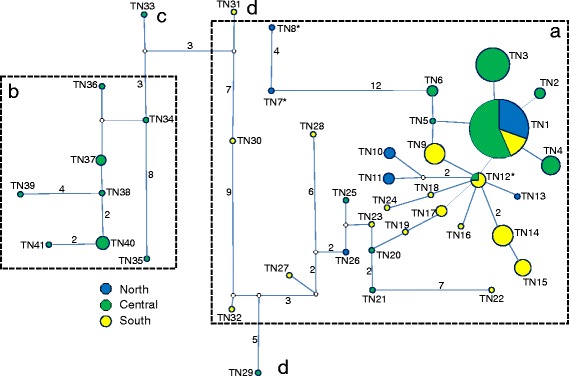
Median-joining network showing the relationships among ND2 haplotypes. Relative haplotype frequencies correspond to haplotype circle size (see Table 1). Numbers on lines connecting haplotypes indicate numbers of mutation changes. Small white circle indicates inferred haplotype. Blue circle: North population, green circle: central population, and yellow circle: south population. **a**, **b**, **c** and **d** are the different amino acids obtained from ND2 (see Fig. 2). *: haplotype linked to outgroup sequences

The obtained ATP6 and ND2 sequences resulted in 34 and 41 haplotypes with 40 and 47 variable sites and with a total length of 399 bp and 348 bp, respectively (see Table 1 for acronyms, numbers of individuals per haplotype, localities, and GenBank accession numbers); for more details of sequence variability see Additional file 1: Table S1 (Supporting information). Both the ATP6 and ND2 sequences translated into four amino acids sequences each, due to two and four non-synonymous positions, respectively (Fig. 2). The phylogenetic relationships among the ATP6 and ND2 haplotype sequences and their translation into amino acid sequences (representing eight different proteins) are shown by the median joining networks in Fig. 3 and Fig. 4. Based on our phylogenetic rooting of the networks by our outgroup taxa, 53.38% of all ATP6 haplotypes were represented by the ancestral protein A, and 89.47% of all ND2 haplotypes were represented by the ancestral protein A. In ATP6 protein B harbored six haplotypes in a star-like evolutionary pattern; one of those modern haplotypes gave rise to protein D, which was the youngest among all four ATP6 proteins. ATP6 protein C consisted of two evolutionarily closely related haplotypes that were derived from a modern haplotype within protein A. In ND2 protein D was derived by two different evolutionary pathways indicating parallel evolution. Protein B and C were derived from evolutionarily young lineages of protein A; they harbored eight and one haplotypes, respectively. Finally, the observed star-like phylogeny of several haplotypes of both mtDNA sequence networks might suggest a rapid population expansion event. Notably, a recent expansion event was also suggested by a significant bimodal distribution of the mtHV-1 haplotypes [23] and by the star-like phylogeny of the transferrin sequences [25] of the same individuals.

**Table 1:** List of the ATP6 and ND2 haplotypes from Tunisia, sample size, geographical origins, and Genbank accession numbers

	ATP6			ND2		
Haploytpe	Sample size	Number and Locality	GenBank Accession	Sample size	Number and Locality	GenBank Accession
TN1	42	BKL(7), BEJ(8), SND(8), KAL(5), NAD(5), STH(5), WES(2), TAT(2),	KX574596	46	BKL(4), BEJ(8), SND(12), KAL(1), DOU(2), NAD(7), STH(7), TAT(3), WES(2)	KX574630
TN2	1	BEJ(1)	KX574597	2	KAL(2)	KX574631
TN3	1	STH	KX574598	16	BKL(15), WES(1)	KX574632
TN4	5	BKL(1), STH(2), BEJ(2)	KX574599	6	SND(3), BKL(3)	KX574633
TN5	1	BEJ(1)	KX574600	1	KAL	KX574634
TN6	1	WES	KX574601	2	NAD(1), KAL(1)	KX574635
TN7	1	BEJ	KX574602	1	BEJ	KX574636
TN8	12	BKL(1), SND(2), NAD(2), DOU(2), TAT(2), CHE(2), KAL(1)	KX574603	1	BEJ	KX574637
TN9	1	SND	KX574604	7	TAT(6), DOU(1)	KX574638
TN10	1	SND	KX574605	2	BEJ(2)	KX574639
TN11	2	CHE(2)	KX574606	2	BEJ(2)	KX574640
TN12	1	SND	KX574607	4	BKL(1), DOU(2), TAT(1)	KX574641
TN13	1	SND	KX574608	1	BEJ	KX574642
TN14	1	BEJ(1)	KX574609	7	TAT(7)	KX574643
TN15	8	DOU(3), TAT(5)	KX574610	4	DOU(3), TAT(1)	KX574644
TN16	1	TAT	KX574611	1	TAT	KX574645
TN17	1	DOU	KX574612	2	DOU(1), TAT(1)	KX574646
TN18	1	BEJ	KX574613	1	TAT	KX574647
TN19	3	BKL(3)	KX574614	1	DOU	KX574648
TN20	2	DOU(1), TAT(1)	KX574615	1	WES	KX574649
TN21	14	CHE(2), DOU(6), BGD(1), NAD(3), WES(2)	KX574616	1	SND	KX574650
TN22	1	SND	KX574617	1	DOU	KX574651
TN23	4	TAT(3), BKL(1)	KX574618	1	DOU	KX574652
TN24	3	SND(2), TAT(1)	KX574619	1	TAT	KX574653
TN25	5	TAT(5)	KX574620	1	CHE	KX574654
TN26	9	NAD, BKL(4), SND(3), CHE(1),	KX574621	1	STH	KX574655
TN27	3	DOU(2), TAT(1)	KX574622	1	BGD	KX574656
TN28	1	TAT	KX574623	1	DOU	KX574657
TN29	1	BKL	KX574624	1	CHE	KX574658
TN30	1	BKL	KX574625	1	WES	KX574659
TN31	1	BKL	KX574626	1	DOU	KX574660
TN32	1	BKL	KX574627	1	DOU	KX574661
TN33	1	BKL	KX574628	1	NAD	KX574662
TN34	1	BKL	KX574629	1	CHE	KX574663
TN35				1	SND	KX574664
TN36				1	CHE	KX574665
TN37				2	CHE(1), SND(1)	KX574666
TN38				1	SND	KX574667
TN39				1	NAD	KX574668
TN40				4	CHE(2), KAL(1), NAD(1)	KX574669
TN41				1	SND	KX574670

For sampling regions acronyms see Fig. 1

### Patterns of positive selection on mtDNA genes

The codon-based tests (estimation of ω per codon) for selection revealed specific codons to be under positive selection (Table 2). The maximum likelihood models that allowed for positive selection fitted to the data of ATP6 significantly better than those that assumed only neutral or deleterious mutations, but for ND2 positive selection was not confirmed by all tests (Table 2, Additional file 2: Table S2). The ω estimates of the model M0 (estimation of ω over all codons) indicated that purifying selection dominated the evolution of both ATP6 (ω = 0.0346) and ND2 (ω = 0.0335). The LRT test for ATP6 indicated that model M2a that allowed for positive selection, fitted better than model M1a that only considered conserved and neutral codon position (Table 2). Estimates using the M2a model suggested that among the two non-synonymous amino-acid changes (sites 72 – A8408G and 76 – G8420A), site 72 in ATP6 was under positive selection. Model M3 that assumed three site classes (ω < 1, ω = 1 and ω > 1), fitted the data better than all the previous models (Table 2). For ND2, in the model comparison (M1a vs. M2a) the alternative model (M1a) was preferred over the null model indicating that substitutions in this gene might be selectively neutral. Moreover, in ND2 the three sites suggested to be under positive selection by Model M2a have a low posterior probability according to the BEB analysis (Table 2). The M2a model for the concatenated genes indicated positive selection for site 72 in ATP6 and sites 16 and 22 in ND2 (Table 2).

**Table 2:** Results of PAML analysis

.*Model*	*ln L*			*ATP6*			*ND2*			*ATP6-ND2*		
	*ND2*	*ATP6*	*ATP6-ND2*	*Parameters*	*Sites* ^*a*^	*p (ΔLRT)*	*Parameters*	*Sites* ^*a*^	*p (ΔLRT)*	*Parameters*	*Sites* ^*a*^	*p (ΔLRT)*
M0 (one ratio)	−1661.369	−1268.911	−4331.728	ω = 0.032			ω = 0.033			ω = 0.034		
M1a (nearly neutral)	−1581.564	−1229.647	−4130.457	p0 = 0.983, p1 = 0.017		<0.001 (M0)	p0 = 0.965, p1 = 0.035		<0.001 (M0)	p0 = 0.975, p1 = 0.024		<0.001 (M0)
M2a (positive selection)	−1578.949	−1219.011	−4107.380	p0 = 0.984, p1 = 0.009, p2 = 0.007, ω2 = 11.336	**72**	<0.001 (M1a)	p0 = 0.965, p1 = 0.009, p2 = 0.026, ω2 = 2.146	16, 22, 25	>0.05 (M1a)	p0 = 0.976, p1 = 0.013, p2 = 0.012, ω2 = 5.092	**72,** 16, **22**	<0.001 (M1a)
M3 (discrete)	−1579.601	−1218.993	−4104.053	p0 = 0.984, p1 = 0.009, p2 = 0.007, ω0 = 0.000, ω1 = 0.89, ω2 = 11.402		<0.001 (M0)	p0 = 0.891, p1 = 0.075, p2 = 0.035, ω0 = 0.000, ω1 = 0.000, ω2 = 1.708		<0.001 (M0)	p0 = 0.932, p1 = 0.056, p2 = 0.012, ω0 = 0.000, ω1 = 0.000, ω2 = 0.356		<0.001 (M0)
M7 (beta)	−1586.014	−1255.405	−4146.712	*p =* 0.005, q = 0.016			*p =* 0.005, q = 0.050			*p =* 0.005, q = 0.039		
M8 (beta and omega)	−1579.100	−1230.106	−4149.305	p0 = 0.985, p1 = 0.015, *p =* 0.005, q = 61.89, ω = 3.406	**72**	<0.001 (M7)	p0 = 0.965, p1 = 0.035, *p =* 0.005, q = 99.000, ω = 1.708	**16,** 22**, 25**	<0.001 (M7)	p0 = 0.976, p1 = 0.024, *p =* 0.005, q = 61.397, ω = 2.392	**72**, **16**, **22**, 25	>0.05 (M7)

Model = name of the model; ***ln L*** = the natural logarithm of the likelihood obtained for every model; Parameters = estimates of ω values and proportion of codons that belong to each ω class; Sites: position in the corresponding gene of each site under positive selection; p(*Δ*LRT) = *P-*values of the log likelihood ratio test for model comparisons, null models are shown in brackets.

^**a**^: BEB: P(ω > 1) > 0.50; P(ω > 1) > 0.95 is shown in bold

The LRT test comparing two models (M7 and M8) that both assume a beta distribution of ω over sites indicated that M8 (that allowed for selection) fitted the data better in both ATP6 and ND2 than M7 (that does not allow for selection). The estimates from M8 indicated that 0.015% (ω = 3.662) and 3.5% (ω = 1.708) of the sites were under diversifying selection in ATP6 and ND2, respectively. For ATP6 one site was identified with a strong signal of positive selection (site 72 – A8408G). While the substitution at position 72 gave rise to the derived protein B from the ancestral protein A, the substitution at position 76 lead to protein C from protein A. For ND2, the same model suggested that three sites were under positive selection (sites 16 – M4171L, 22 – H4189Y, and 25 – L4199P) with a posterior probability of >95% for sites 16 and 25, and a posterior probability of >90% for site 22 (Additional file 2: Table S2). Substitutions at all those three sites with site 23 (L4192F) lead to the modern protein B from the ancestral protein A (see Fig. 2, Fig. 4). Although, model 8 confirmed the detected sites under positive selection for the concatenated genes, LRT comparison (M7 vs M8) suggests that M7 was preferred to the alternative model (M8).

The following PAML results were confirmed by tests provided by the web server DATAMONKEY: 1) site 72 of ATP6 was identified to be under “episodic diversifying selection” with the MEME (*p <* 0.05) method. Notably, site 72 at the ATP6 locus produced the amino acid difference between the two main proteins (A and B) with an overall frequency of occurrence of 97.7%; 2) various other sites of ATP6 were identified as negatively selected, and depending on the methods used their numbers ranged from 7 (SLAC) to 42 (REL); 3) for ND2, no site showed any evidence of positive selection; however, 20 (SLAC) to 43 (REL) sites were identified to be negatively selected; 4) finally, site 72 (ATP6) was confirmed by FEL (*P =* 0.016), MEME (*P =* 0.02) and REL (Bayes factor = 374.32) when applied to the concatenated genes and site 22 (ND2) was confirmed by the REL (Bayes factor = 1680.55) test.

### Genetic differentiation of mtDNA and microsatellite loci

The AMOVA results for the mtDNA sequences and the microsatellite genotypes on partitioning of genetic diversity among the three regions (NT, CT, ST) are summarized in Table 3. For microsatellites only 2.49% of the overall relative genetic variability was partitioned into the three regions. Similarly, only 5.07% of HV-1 sequence variability was due to partitioning among the three regions, when calculated from haplotype frequencies, and 17.43% of the HV-1sequence variability was partitioned among regions, when calculated by means of the distance method. The partitioning of variability of the ATP6 sequences among regions amounted to 19.02% and 13.23%, when calculated from haplotype frequencies and pairwise sequence distances, respectively. A similar level of partitioning was observed for ND2 (19.42% and 6.36%). The AMOVA models based on translated amino-acid frequencies in the three regions resulted in an F_ST_ of 42.66% for ATP6 proteins and an F_ST_ of 8.31% for the ND2 proteins. Leaving out the two non-synonymous nucleotide positions (A8408G and G8420A) of the ATP6 sequences resulted in distinctly lower relative genetic differentiation (6.28% and 8.06%) than that for the complete sequences (19.02% and 13.23%) (for details see Table 3). Pairwise *F*_ST_values of relative genetic differentiation between regions (Additional file 3: Table S3) reached a maximum of 6.15% (CT/ST) for mtHV1sequences and 4.1 for microsatellites (NT/CT). The respective values were clearly higher for ATP6 (15.46%–23.64%) and ND2 (14.05%–26.02%) sequences.

**Table 3:** Partitioning of genetic variation (AMOVAs) as calculated from the different markers at two levels

		Source of variation		df	% of variation
*ATP6*	All sites	Among regions	F_ST_	2	19.02*
		Within regions		130	80.98*
		Among regions	d	2	13.23*
		Within regions		130	86.77*
	Syn. sites	Among regions	F_ST_	2	6.28*
		Within regions		130	93.72*
		Among regions	d	2	8.06*
		Within regions		130	91.94*
	protein frequencies	Among regions	F_ST_	2	42.66*
		Within regions		130	57.34*
*ND2*	All sites	Among regions	F_ST_	2	19.42*
		Within regions		130	80.58*
		Among regions	d	2	6.36*
		Within regions		130	93.64*
	Syn. sites	Among regions	F_ST_	2	8.91*
		Within regions		130	91.09*
		Among regions	d	2	6.08*
		Within regions		130	93.92*
	protein frequencies	Among regions	F_ST_	2	8.31*
		Within regions		130	91.69*
mtHV1		Among regions	F_ST_	2	5.07*
		Within regions		130	94.93*
		Among regions	d	2	17.43*
		Within regions		130	82.57*
Microsatellites		Among regions	F_ST_	2	2.49*
		Within regions		130	97.51*

For ATP6 and ND2, AMOVAs were calculated based on sequences including all nucleotides, on sequences including synonymous (syn.) sites only, and in protein frequencies. Asterisks denote values significantly (*p <* 0.05 after Bonferroni corrections) higher than zero

### Protein frequencies and co-occurrence of proteins

Overall frequencies of the four ATP6 proteins (A, B, C, D) amounted to A = 53.4%, B = 44.3%, C = 1.5%, D = 0.8% and frequencies of the two most prevalent proteins A and B varied significantly between regions in all pairwise comparison (NT vs. CT: chi^2^ = 15.6, *p <* 0.0005; CT vs. ST: chi^2^ = 20.2, *p <* 0.0005; NT vs. ST: chi^2^ = 47.2, *p <* 0.0005). Overall frequencies of the four ND2 proteins (A, B, C, D) amounted to A = 89.5%, B = 8.3%, C = 0.8%, and D = 1.5%. Frequencies of the two most prevalent ND2 proteins A and B, however, differed significantly only between regions CT and ST (chi^2^ = 6.5, *p =* 0.015, two-sided test), when accounting for multiple tests.

The Pearson φ coefficient for association of the two most prevalent ATP6 and ND2 proteins A and B, respectively, indicated a week (φ = 0.366) but significant (*p =* 0.002) positive association between the ancestral ATP6 A and the derived ND2 B proteins and *vice versa* in individual hares from the CT region.

The SPIDERMONKEY run indicated a significant interaction (*p =* 0.5) between positions 72 at ATP6 and 22 at ND2 with a posterior probability of 0.27 for site 72 being conditionally dependent on site 22, and with a posterior probability of 0.23 for site 22 being conditionally dependent on site 72.

### Models of ATP6 and ND2 protein occurrence

The logistic glm runs and the model averaging statistics indicated a significant positive effect of “temp“ (mean annual temperature of the year)on the presence of the ancestral ATP6 protein A (i.e., a higher likelihood of presence at higher “temp“) and a negative effect on the modern ATP6 protein B, i.e., higher likelihood of presence at lower “temp“, independent of geographical position of the sampling site, precipitation, and the ND2 protein variant co-occurring in the same mtDNA. However, there was also a significant negative effect of ND2 proteins on the occurrence of the ATP6 A protein, independent of the other fixed factors/variables. Relative variable importance values (RVI) of precipitation and latitude were not much above 0.5, indicating no clear effects on the presence of ATP6 protein A or B (Table 4).

**Table 4:** Summary results of the logistic linear model of occurrence of ATP6 protein A or B after model averaging

variable	coefficient	p	RVI
**nd2ab**	−2.725	**0.0136**	**0.99**
**temp**	−0.8661	**0.0194**	**0.88**
prec	5.806e-03	0.1296	0.59
latitude	7.374e–05	0.1642	0.56
longitude	−1.842e–05	0.6670	0.28

All model-averaged variable coefficients, associated values of significance (p) and values of relative variable importance (RVI) are listed; nd2ab—presence of ND2 A or B protein in the same mtDNA molecule, respectively, temp—mean annual temperature, prec—mean annual precipitation, latitutde/longitude—geographical latitude/longitude of sampling location, p—significance value. Variables with RVI values > 0.7 are considered of significant importance; variables in bold have significant effects on the presence of ATP6 A or B proteins

The logistic glm runs and the associated model averaging statistics for the ND2 proteins A and B indicated a highly significant positve effect of “mintemp” (mean minimum temperature of the coldest month of the year) on the occurrence of the ancestral ND2 protein A and conversely a negative effect on the occurrence of the derived ND2 protein B, independent of geographical location, annual precipitation, and the ATP6 protein co-occurring in the same mtDNA. In addition, there was a significant negative effect of the co-occurring ATP6 protein variant, i.e., the presence of the ancestral ND2 protein A was favoured when the modern ATP6 protein B was co-occurring in the same mtDNA and *vice versa*. Apart from that finding there was a significant negative longitude effect, independent of all other factors (Table 5).

**Table 5:** Summary results of the logistic linear model of occurrence of ND2 protein A or B

variable	coefficient	p	RVI
**atp6ab**	−2.958	**0.0154**	**0.99**
**min. temp**	2.566	**0.0043**	**0.98**
**longitude**	−2.771e–04	**0.0101**	**0.97**
latitude	−5.351e–05	0.5235	0.31
prec	−7.181e–04	0.9454	0.26

All model-averaged variable coefficients, associated values of significance (p) and values of relative variable importance (RVI) are listed; atp6ab—presence of ATP6 A or B protein in the same mtDNA molecule, respectively, min. temp—mean minimum temperature of the coldest month of the year, prec—mean annual precipitation, latitutde/longitude—geographical latitude/longitude of sampling location, p—significance value. Variables with RVI values > 0.7 are considered of significant importance; variables in bold have significant effects on the presence of ND2 A or B proteins

## Discussion

Several attempts have been made to understand positive (diversifying) selection on mitochondrial (mt) OXPHOS genes in the context of geographical and environmental characteristics such as geographical clines [53–55], altitude [56], subterranean habitat [57], and particularly in relation to ambient temperature variation (e.g., [8, 13, 14, 55]). Given the paramount role of mitochondria for cellular energy production, selective effects of ambient temperature on the mitochondrial genes encoding for the OXPHOS process is very plausible. Positive selection on those genes may act particularly under heterogenous environments in terms of time and space. Effects of ambient temperature on genetic variation in natural populations may, however, be obscured by other environmental factors that vary for instance geographically [58] or by historical population genetic changes, particularly, if ambient temperature effects are not much pronounced. In addition, coevolution effects among mt OXPHOS genes on the one hand and within the mitonuclear gene complex responsible for the OXPHOS process on the other can be expected to blur positive selection signals at single sites (e.g, [14, 15]). Similarly, epistatic effects may produce certain clusters of non-synonymous sites within and among proteins [59], particularly in functionally important and coevolving genes, as in the mt OXPHOS complex. Such functional clusters of coevolving sites might also reduce the chance of detecting positive selection signals, if e.g., several of those sites are under purifying selection.

Here we studied amino acid sequence variation at two mt loci of cape hares (*Lepus capensis*) from Tunisia, across a steep ecological gradient within comparatively short geographic distances (ca. 450 km as the craw flies) from near to the northern Mediterranean seaboard to the arid Sahara zone in the south. In spite of marked variation of external phenotypes (particularly body size and coat colouration), all hares from our study range are genetically and phylogenetically closely related as revealed by our microsatellite and mtHV1 sequence data (see also [31, 60]). In fact, our current AMOVAs of variation of nDNA and mtDNA indicated a low level of partitioning of genetic variability across the whole study area that was well within the range commonly found among populations of terrestrial mammal species; also, our earlier phylogenetic mtHV1 sequence analysis indicated no major phylogenetic gap among the Tunisian hares [31, 60]. Notably, those earlier phylogenetic and microphylogeographic results were based on the same individuals that we used for the presently obtained amino acid data. Hence, the same individual mitochondria populations were used both for the microphylogenetic/phylogeographic information and the amino acid data. The phylogenetically rather homogenous gene pool and the shallow population structure of Tunisian hares that display otherwise several external phenotypes provide a good precondition to detect positive selection related to environmental/climatic variation, specifically ambient temperature and precipitation.

Indeed, we have proved positive selection to occur at the ATP6 locus in the Tunisian hares that translates into four proteins with two of them at a very high combined frequency of occurrence. Moreover, the two most prevalent proteins at that locus are distributed at significantly varying frequencies across the study area with a higher frequency of the ancestral protein variant in the warmer southern Sahara region and the derived protein at a higher frequency in the cooler Mediterranean climate province in the north. Concordantly, we found a significant effect of mean annual temperature on the presence of the ancestral or the most prevalent derived ATP6 protein, with the occurrence of the ancestral protein variant being favoured at higher temperature and the most frequent derived protein being favoured at lower temperatures. Apparently, this seems to be a general effect of ambient temperature as it is independent of the geographic sampling location (geographical longitude and latitude).

For ND2, the second locus under study, a signal of positive selection was also obtained by several of the used codon-based tests. Accordingly, the ND2 protein frequencies differ also significantly between the three discriminated ecological regions. Moreover, the significant effect of ambient temperature on the occurrence of the two most frequent ND2 protein variants independent of geographic sample locations corroborates our site based-test results. Remarkably, occurrence of the two most frequent ND2 protein variants, the ancestral and the most frequent derived variant, is influenced by mean minimum temperature of the coldest month (February) of the year, rather than by mean annual temperature (which is the case for the two most frequent ATP6 protein variants). This may point towards a combined and more complex effect of various aspects of ambient temperature acting on diverse mtOXPHOS genes of single hares in a co-adaptive way. The significant effect of geographical longitude on the occurrence of the ND2 protein variants might indicate a climate aspect related to the distance from the seabord, such as seasonal humidity, that was, however, not investigated in our current analysis.

Finally and most notably, we obtained a significant coevolution signal for the two loci under study and detected diversifying (positive) selection on the composite haplotype combining the ancestral ATP6 protein and the most frequent derived ND2 protein and the composite haplotype combining the most frequent derived ATP6 protein and the ancestral ND2 protein. These two protein combinations were found in 51.1% of all studied hares but did not occur in the Sahara region of our study, because the most prevalent derived ND2 protein occurred only in the semiarid central Tunisian and the Mediterranean north Tunisian regions. The predominant occurrence of the most prevalent derived B protein at this locus in central Tunisia may suggest recent evolution there or recent immigration from western ranges (Algeria) into central Tunisia. Nevertheless, the favoured co-occurrence of either the ancestral ATP6 and the most prevalent derived ND2 proteins or the most prevalent derived ATP6 and the ancestral ND2 proteins—hence, the favoured occurrence of either composite haplotype—independent from geographical coordinates of sample locations and independent of climate factors can be interpreted as positive selection favouring either of the two composite haplotypes. That latter finding, together with the significant variation of protein frequencies across the three climate provinces in Tunisia, as well as the significant effects of mean annual temperature on ATP6 protein occurrence and mean minimum temperature of the coldest month of the year on ND2 protein occurrence independent from geographical location strongly suggests that the observed sequence patterns at the two loci are indeed due to positive selection, rather than to relaxation of selective constraints as e.g. discussed by [61–63].

Remarkably, the currently detected sites proved to be under positive selection and being affected by ambient temperature were not found to be under positive selection by [14], who tested for positive selection signals in entire mitogenomes of eleven *Lepus* species, which was probably due to their small number of haplotypes (see e.g., [44, 64]. The latter authors also used advanced remote homology detection methods to build 3D models, predict ligand binding sites, and analyze possible amino acid variants resulting from nonsynonymous mutations across the entire mitogenomes. However, they failed to identify possible effects on single variants that were proved to be under positive selection. They suggested that the focus of selection might lie on complex interactions with nuclear encoded peptides.

Nevertheless, in mouse cells a single nucleotide polymorphism in the *mttRNA-Arg* gene produces differences in the OXPHOS performance [65], and single or few non-synonymous mitochondrial substitutions can have strong negative effects on human health or metabolic performance. According to [66–68] single mitogenome mutations are responsible for or increasing the chance of developing a broad range of metabolic and degenerative diseases, cancer, and aging due mainly to energetic dysfunction (see e.g., [69, 70]). A total of 308 mitogenome mutations are responsible for or associated with different diseases in humans (see MITOMAP: A Human Mitochondrial Genome Database; http://www.mitomap.org; last accessed 15 March 2016 [71]). For ATP6, 24 mutations are associated with twenty different diseases in humans; all these mutations represent single nucleotide substitution, except for the occurrence of one deletion. For instance, the mutation A8836G changes Methionine to Valine and is associated with the LHON (Leber Hereditary Optic Neuropathy) disease. Noteably, the position 72 at the ATP6 locus in our study that is under positive selection represents such a mutation (A8408G) resulting in the same amino acid change from Methionine to Valine. For ND2, 16 mutations are reported to be associated with a total of 19 different human diseases; except for two frame shift mutations, all are single nucleotide substitution.

In spite of no immediate functional information on the presently found ATP6 and ND2 protein polymorphisms, their adaptive significance is suggested by 1) the general role of the proteins in the OXPHOS process of cellular energy and heat production, 2) the varying distribution of the proteins across the different climate zones of our study region, and 3) the effects of ambient temperature on the occurrence of the protein variants. Remarkably, the observed ambient temperature effects on the occurrence of the two most frequent protein variants at each locus are independent of geographic sampling localities. This suggests direct involvement of ambient temperature in the selection of these proteins, rather than potential historic population genetic reasons. Our finding that the currently revealed most prevalent ATP6 protein variants are affected by mean annual temperature and the occurrence of the most prevalent ND2 protein variants in our study are affected by mean minimum temperature of the coldest month of the year may suggest a combined selective effect of even more (currently not considered) ambient temperature characteristics in a more complex way on the cellular energy production (e.g., for basal metabolic rate or regulation of body temperature). Selection coefficients of protein variants of other mt or nuclear OXPHOS genes may vary with temperature (climate) characteristics currently not studied, and their likely coevolution to optimize the OXPHOS process in relation to climatic variation is far from being understood.

In brown hares (*Lepus europaeus*), a species phylogenetically closely related to the currently studied cape hares or even conspecific with them [26, 27], particularly leverets and young during their early period of growth are supposed to exhibit increased mortality under low ambient temperature, rainy and windy weather, in addition to other factors (e.g. [72, 73]). High energy costs of young hares for thermoregulation during their early period of growth were pointed out by [74]. Selection on different mt or nOXPHOS genes may vary in the course of the onthogenetic development or across different climatic regions, particularly under additional ecological circumstances, such as infection with diverse parasites or pathogens that increase energy demands for the immune reaction. Therefore, positive selection on spatiotemporally varying combinations of genes is conceivable within one and the same species operating during critical phases of the ontogeny or under varying ecological/climatic environments. As demonstrated by [75] resting metabolic rate of hares (considered *Lepus capensis*) from the Negev Desert of Israel had a resting metabolic rate of only 61% of brown hares from south France. The latter are currently classified as *L. europaeus*. Similar physiological analyses are to date not available for the currently studied hares, but similar climatic characteristics as for the hares from the Negev Desert on the one hand and in south France on the other may suggest similarly strong physiological adaptations in hares from the different climate zones of Tunisia. Ideally, experimental (e.g., translocation experiments) assession of selection on various mtOXPHOS genes and concomitant determination of physiological parameters such as resting or field metabolic rates of hares with different mtOXPHOS proteins should complement our current results.

## Conclusion

Several evidences obtained in the current study strongly suggest an adaptive significance of the amino acid changes in both the ATP6 and the ND2 loci: 1) the important role of the proteins in cellular metabolism and cellular energy and heat production 2) the significantly varying distribution of the proteins across the different climate zones of our study region, despite little geographical differentiation in non-coding d-loop sequences of the same mitogenomes; 3) the significant signals of positive selection on those sites that translate into the most prevalent proteins at both loci; and 4) the significant effects of ambient temperature (mean annual temperature and mean minimum temperature of the coldest month of the year) on the occurrence of the protein variants. This might suggest strong physiological adaptations (i.e., cellular energy production) in hares from the different climate zones of Tunisia. However, experimental assession of selection on various mtOXPHOS genes and concomitant determination of physiological parameters is necessary to complement our current results.

## Additional files

Additional file 1: Table S1. Basic sequence statistics for the three mitochondrial regions (ND2, ATP6, mtHV1) as calculated by the software DNASP (Librado & Rozas 2009). S: number of segregating (polymorphic) sites; h: haplotype diversity; *π:* nucleotide diversity; k: average number of nucleotide differences; D (Tajima 1989); D (Fu & Li 1993); F (Fu & Li 1993), and F (Fu 1997). (DOCX 14 kb)
Additional file 2: Table S2. Posterior probabilities (PP) for the currently studied sites following the BEB approach in PAML. These values were reported for single gene analyses and the concatenated genes and for both models allowing positive selection. P*P >* 0.95 are grey shaded. (DOCX 11 kb)
Additional file 3: Table S3. Pairwise F_ST_ values for the three regions as obtained from the different markers. Upper diagonal, F_ST_ values (based on haplotype frequencies) as calculated from ATP6, ATP6 synonymous (syn.) positions only, ND2, ND2 syn. positions only, mtHV1, and from the microsatellite data. Lower diagonal, F_ST_ values (based on the distance method) as calculated from ATP6, ATP6 syn. positions, ND2, ND2 syn. positions and mtHV1, with 95% CI indicated between parentheses. Significance levels, *: *P* < 0.05, **: *P* < 0.01, ***: *P* < 0.001. (DOCX 13 kb)
