# Supplementary material for: Selection on the mitochondrial ATP synthase 6 and the NADH dehydrogenase 2 genes in hares (Lepus capensis L., 1758) from a steep ecological gradient in North Africa
Source: BMC Evol Biol. 2017 Feb 7;17:46. doi: 10.1186/s12862-017-0896-0 (PMC5297179; doi:10.1186/s12862-017-0896-0)
Supplement: Additional file 1: Table S1. — Basic sequence statistics for the three mitochondrial regions (ND2, ATP6, mtHV1) as calculated by the software DNASP (Librado & Rozas 2009). S: number of segregating (polymorphic) sites; h: haplotype diversity; π: nucleotide diversity; k: average number of nucleotide differences; D (Tajima 1989); D (Fu & Li 1993); F (Fu & Li 1993), and F (Fu 1997). (DOCX 14 kb) [file 12862_2017_896_MOESM1_ESM.docx]

**Additional file 1: Table S1** Basic sequence statistics for the three mitochondrial regions (ND2, ATP6, mtHV1) as calculated by the software DNASP (Librado & Rozas 2009). S: number of segregating (polymorphic) sites; h: haplotype diversity; *π:* nucleotide diversity; k: average number of nucleotide differences; D (Tajima 1989); D (Fu & Li 1993); F (Fu & Li 1993), and F (Fu 1997)

| ***Domain*** | ***region*** | ***S*** | ***h*** | ***π*** | ***k*** | ***Tajima's D*** | ***Fu and Li's D*** | ***Fu and Li's F*** | ***Fu's F*** |
| --- | --- | --- | --- | --- | --- | --- | --- | --- | --- |
| ***ATP6*** | ***All*** | 40 | 0.869 | 0.011 | 4.512 | -1.302 | -0.019 | -0.663 | -13.665*** |
|  | ***North*** | 8 | 0.696 | 0.005 | 2.134 | -0.050 | -0.433 | -0.373 | -0.724 |
|  | ***Central*** | 34 | 0.814 | 0.012 | 4.755 | -1.158 | -0.906 | -1.198 | -3.508* |
|  | ***South*** | 20 | 0.881 | 0.010 | 4.165 | -0.417 | -0.377 | -0.461 | -1.657 |
| ***ND2*** | ***All*** | 47 | 0.859 | 0.027 | 9.270 | 0.238 | 2.214** | 1.665 | -8.04*** |
|  | ***North*** | 41 | 0.632 | 0.021 | 7.265 | -1.341 | -0.951 | -1.255 | 2.677 |
|  | ***Central*** | 42 | 0.818 | 0.031 | 11.113 | 0.920 | 1.795** | 1.746* | 0.564 |
|  | ***South*** | 33 | 0.906 | 0.016 | 5.593 | -1.006 | 0.238 | -0.219 | -2.391* |
| **mtHV1** | ***All*** | 75 | 0.966 | 0.027 | 12.570 | -0.483 | -0.175 | -0.371 | -2.014 |
|  | ***North*** | 35 | 0.960 | 0.026 | 11.711 | 0.530 | 0.760 | 0.807 | -1.872 |
|  | ***Central*** | 41 | 0.913 | 0.020 | 9.310 | 0.242 | -0.050 | 0.075 | -0.433 |
|  | ***South*** | 58 | 0.966 | 0.032 | 14.788 | 0.192 | 0.724 | 0.640 | -1.982 |

*p < 0.05; **p < 0.01; ***p<0.001
