# Supplementary material for: Selection on the mitochondrial ATP synthase 6 and the NADH dehydrogenase 2 genes in hares (Lepus capensis L., 1758) from a steep ecological gradient in North Africa
Source: BMC Evol Biol. 2017 Feb 7;17:46. doi: 10.1186/s12862-017-0896-0 (PMC5297179; doi:10.1186/s12862-017-0896-0)
Supplement: Additional file 2: Table S2. — Posterior probabilities (PP) for the currently studied sites following the BEB approach in PAML. These values were reported for single gene analyses and the concatenated genes and for both models allowing positive selection. PP > 0.95 are grey shaded. (DOCX 11 kb) [file 12862_2017_896_MOESM2_ESM.docx]

**Additional file 2: Table S2** Posterior probabilities (PP) for the currently studied sites following the BEB approach in PAML. These values were reported for single gene analyses and the concatenated genes and for both models allowing positive selection. PP>0.95 are grey shaded.

|  | **ATP6** | | **ND2** | | **AT6-ND2** | |
| --- | --- | --- | --- | --- | --- | --- |
| Site\Model | **M2** | **M8** | **M2** | **M8** | **M2** | **M8** |
| **72** | 1 | 1 |  |  | 1 | 1 |
| **16** |  |  | 0.879 | 0.980 | 0.878 | 0.999 |
| **22** |  |  | 0.805 | 0.937 | 0.992 | 1 |
| **25** |  |  | 0.798 | 0.967 |  | 0.661 |
