# Supplementary material for: Selection on the mitochondrial ATP synthase 6 and the NADH dehydrogenase 2 genes in hares (Lepus capensis L., 1758) from a steep ecological gradient in North Africa
Source: BMC Evol Biol. 2017 Feb 7;17:46. doi: 10.1186/s12862-017-0896-0 (PMC5297179; doi:10.1186/s12862-017-0896-0)
Supplement: Additional file 3: Table S3. — Pairwise FST values for the three regions as obtained from the different markers. Upper diagonal, FST values (based on haplotype frequencies) as calculated from ATP6, ATP6 synonymous (syn.) positions only, ND2, ND2 syn. positions only, mtHV1, and from the microsatellite data. Lower diagonal, FST values (based on the distance method) as calculated from ATP6, ATP6 syn. positions, ND2, ND2 syn. positions and mtHV1, with 95% CI indicated between parentheses. Significance levels, *: P < 0.05, **: P < 0.01, ***: P < 0.001. (DOCX 13 kb) [file 12862_2017_896_MOESM3_ESM.docx]

**Additional file 3: Table S3** Pairwise F_ST_ values for the three regions as obtained from the different markers. Upper diagonal, F_ST_ values (based on haplotype frequencies) as calculated from ATP6, ATP6 synonymous (syn.) positions only, ND2, ND2 syn. positions only, mtHV1, and from the microsatellite data. Lower diagonal, F_ST_ values (based on the distance method) as calculated from ATP6, ATP6 syn. positions, ND2, ND2 syn. positions and mtHV1, with 95% CI indicated between parentheses. Significance levels, *: *P* < 0.05, **: *P* < 0.01, ***: *P* < 0.001

|  | **NT** | **CT** | **ST** |
| --- | --- | --- | --- |
| **NT** | - | 0.206*  0.017  0.141*  0.058**  0.041*  0.012* | 0.155*  0.076**  0.221*  0.141***  0.035*  0.027* |
| **CT** | 0.052*(-0.004-0.133)  0.006 (-0.007-0.023)  0.022 (0.004-0.032)  0.018 (0.001-0.031)  0.071* (0.039-0.110) | - | 0.236*  0.082***  0.260*  0.088***  0.061*  0.028* |
| **ST** | 0.267***(0.059-0.504)  0.101***(0.043-0.147)  0.039 (0.007-0.064)  0.044 (0.007-0.127)  0.114***(0.078-0.163) | 0.132***(0.051-0.193)  0.110***(0.036-0.165)  0.093**(0.007-0.064)  0.090**(0.036-0.018)  0.241***(0.178-0.284) | - |
